# Vertical and Transverse Management with Transpalatal Arches in an Adult with Class III Malocclusion

**DOI:** 10.1155/2017/4062105

**Published:** 2017-05-31

**Authors:** R. M. Yañez-Vico, M. Cadenas de Llano Perula, E. Solano-Reina

**Affiliations:** Stomatology Department, School of Dentistry, University of Seville, C/Avicena sn, 41009 Seville, Spain

## Abstract

The transpalatal arch might be one of the most common intraoral auxiliary fixed appliances used in orthodontics in order to provide dental anchorage. The aim of the present case report is to describe a case in which an adult patient with a tendency to class III, palatal compression, and bilateral posterior crossbite was treated with double transpalatal bars in order to control the torque of both the first and the second molars. Double transpalatal arches on both first and second maxillary molars are a successful appliance in order to control the posterior sectors and improve the torsion of the molars. They allow the professional to gain overbite instead of losing it as may happen with other techniques and avoid enlarging of Wilson curve, obtaining a more stable occlusion without the need for extra help from bone anchorage.

## 1. Introduction

The transpalatal arch might be one of the most common intraoral auxiliary fixed appliances used in orthodontics in order to provide dental anchorage. When placed passive, it brings three-dimensional control; it helps to maintain transverse dimensions of the posterior sector during treatment, maintain spaces during movement of the lateral sectors, and, if placed active, can produce molar rotation and uprighting [1]. The function of the transpalatal arch is to avoid buccal or palatal tipping of the molars during the application of an intrusive force to the incisors; it keeps the position of the molars and assures the effect of the force to expand homogeneously when the lateral sectors are moving. Unlike other appliances for the correction of maxillary molar excessive eruption (head gear, chin cup, among others), the transpalatal arch is a fixed appliance that avoids patient cooperation to be a crucial point on which satisfactory results may depend [2].

The purpose of this paper is to report a case in which an adult patient with a tendency to class III, palatal compression, and bilateral posterior crossbite was treated with double transpalatal bars in order to control the torque of both the first and the second molars.

## 2. Diagnosis and Etiology

**Figure 1:**
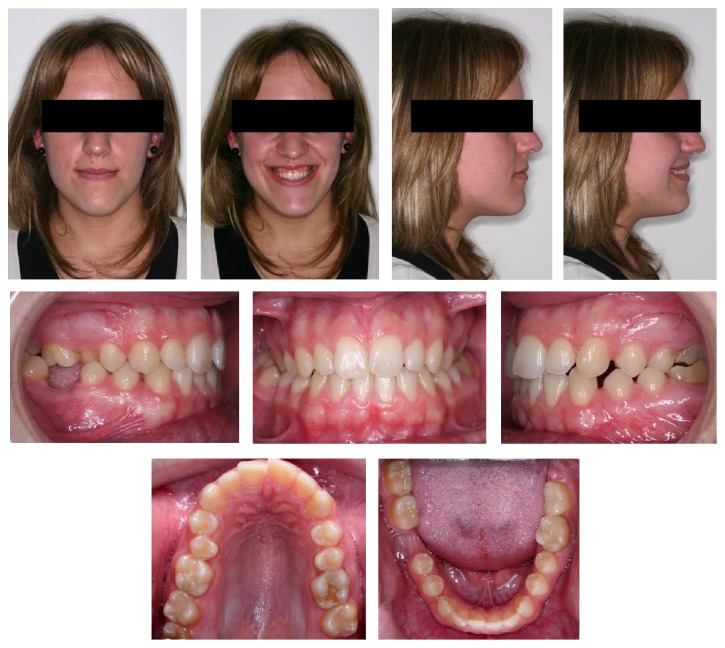
Intra- and extraoral initial records.

A woman aged 20 years and 5 months came to the Orthodontics Department of our Dentistry School (University of Seville, Spain) with the chief complaint of “crossbite.” The patient experienced extreme pain sensibility and dental scare due to previous negative dental experiences and traumatic lesions during childhood [3–5]. She had a straight profile, bilateral molar angle class III, and canine angle class I left II right. She was missing the right mandibular molar, and the midlines were not coincident (the lower midline was 1 mm more to the left than the upper one). She had bilateral posterior crossbite, a 1 mm overjet, and 0.5 mm overbite and no crowding (arch-length discrepancies: maxilla, 0 mm; mandible, +10 mm). No signs of temporomandibular problems were found, but the intermolar width was decreased in the upper arch (37 mm) and there was palatal compression [6] (Figure 1).

**Figure 2:**
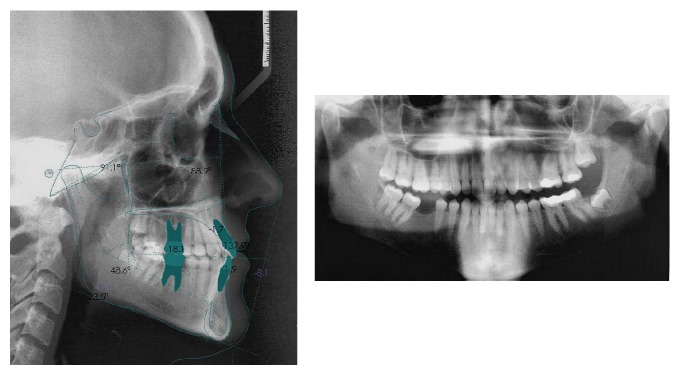
Lateral and panoramic initial radiographies.

The cephalometric analysis showed that the patient was mesofacial (gonial angle 120.4°, mandibular plane angle 23.5°), skeletal class I (convexity, 1.7 mm), having a class III occlusal plane angle (83.9°) and a normal lower facial height of 48.6°. Tooth axial inclination was 60.1° for the maxillary incisor and 77.7° for the mandibular incisor (both lingually inclined) and a normal interincisal angle (137.9°) (Figure 2).

## 3. Treatment Objectives

The treatment objectives were to

- obtain molar and canine bilateral class I,
- achieve coincident midlines,
- solve the posterior crossbite,
- expand the upper arch and correct the torque of the molars,
- open the space for the prosthetic replacement of the right mandibular molar with an implant,
- improve the amount of overbite.

## 4. Treatment Alternatives

As the patient was an adult, our treatment choice was to expand the upper arch with a removable plate with an expansion screw and a bite plane and fixed orthodontic appliances in order to decompensate the lower arch and reopen the space for the right first mandibular molar that would be replaced with an implant. Double transpalatal bars were also used in order to modify the torque of the upper molars and create overbite; as control of the posterior sectors in this case can be difficult, it is not unusual to end up with excessive Wilson curve.

Bone anchorage, such as miniscrews, could have been used as well, but our intention was to solve the case in the simplest way possible.

## 5. Treatment Progress

**Figure 3:**
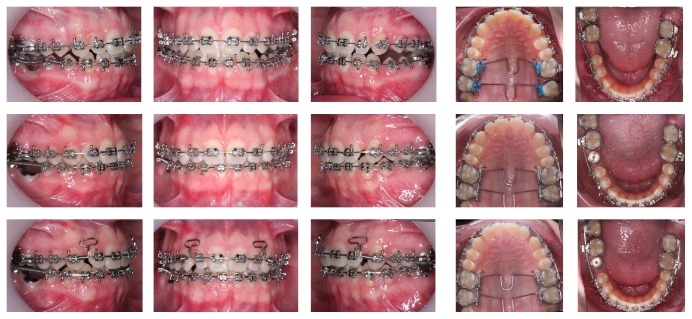
Intraoral treatment progress records.

The active removable expansion plate was placed for six months; it had a bite plane from the first premolars to the third left molar and the second right molar (the third right molar was not in the mouth). The bite plane was reduced in every appointment in order to slightly extrude incisors and intrude molars at the same time that we produced expansion that would solve the bilateral crossbite (Figure 3).

After three months, our goals regarding expansion were achieved and the patient had the plate for three more months in order to consolidate the results. After that, fixed multibracket appliances were placed, together with the transpalatal bars, on both first and second maxillary molars. They were activated in order to reach molar derotation and uprighting, correcting the torque of the molars, thus gaining overbite (Figure 3).

**Figure 4:**
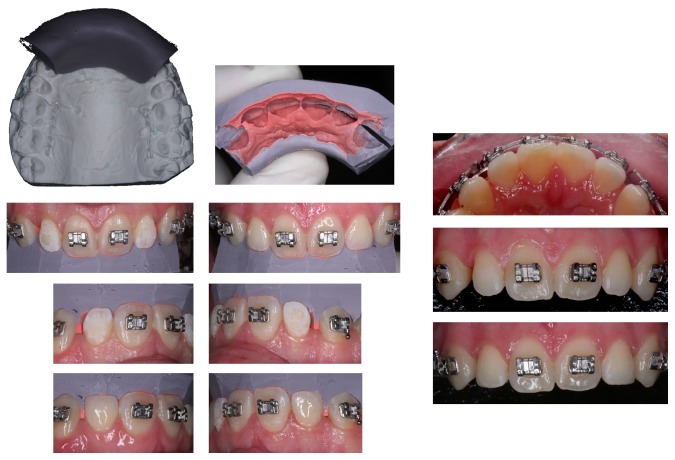
Detailed process of the final lateral esthetic restoration. Note the remaining space to be retracted with a T loop TMA archwire.

The usual sequence of arches was followed, and an open coil spring was placed between the mandibular right second premolar and second molar, to start with space reopening. A TMA arch [7] with “T” loops was placed on the upper arch in order to put the anterior front together, and once on their correct position, a closed coil spring was placed distal to both “T” loops to maintain the space for the prosthetic reconstruction of the lateral, whose size was too small (Figure 4).

In the meanwhile, the implant surgery for the right first mandibular molar was performed and a provisional crown was placed so that it could be used to achieve the uprighting of the lower second right molar. The roots were checked radiographically to be parallel and coordination of the arches and final offsets and compensation bends were made (Figure 3).

## 6. Treatment Results

At the end of the treatment, bilateral molar and canine class I were achieved, as well as 3 mm overbite and 0.5 mm overjet. Upper intermolar width went from 37 mm at the beginning of the treatment to 45 mm when finished.

**Figure 5:**
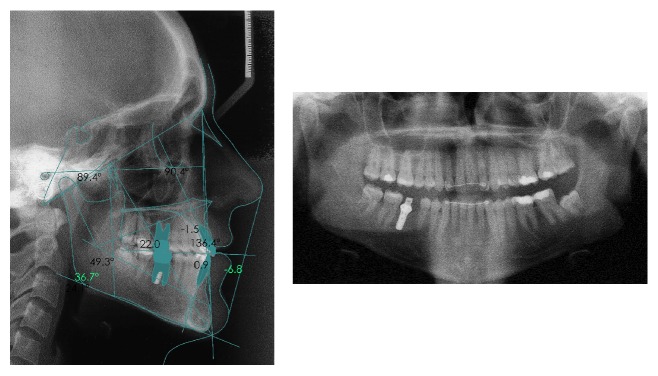
Lateral and panoramic final radiographies.

Cephalometrically, the convexity did not vary significantly, as it was also previously normal (from −1.7 to −1.5 mm), but the occlusal plane angle went from class III (83.9°) to class I (89.2°). Both maxillary and mandibular incisors were lingually inclined at the beginning of the treatment and they were both inclined buccally (from 60.1° to 62.6° and 77.7° to 73.8°, resp.). On the superimpositions, we can see that the bigger changes were performed on the position of the molars, over all the upper ones, whose torque was corrected (Figure 5).

The smile was then wider, without black lateral corridors due to the expansion performed, midlines are coincident, the lip level matched the gingival level of the central upper incisors, and an average of 3 mm of gum is shown when smiling. The size of the maxillary laterals was also improved which adds harmony to the smile. The profile has been also slightly improved, due to the support that the more buccally inclined incisors bring to the lips.

**Figure 6:**
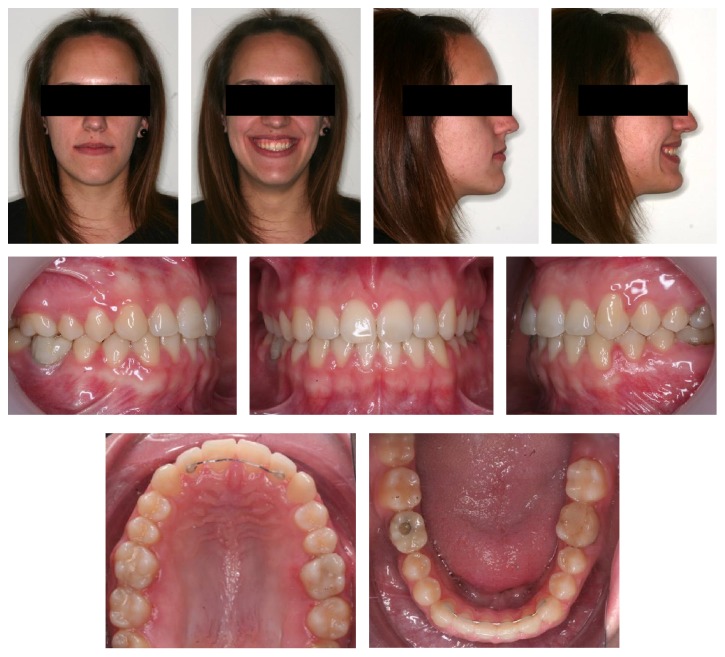
Intra- and extraoral final records.

Functionally, we made sure that both lateral and protrusive jaw movements were correctly done, without improper contacts of the rest of the teeth, and no signs of temporomandibular problems, pain, or distress were found. Fixed retention bars were placed from lateral to lateral in the upper arch and from canine to canine on the lower arch in order to avoid relapse (Figure 6); nevertheless, final decision for removing the fixed retention was considered due to periodontist recommendation based on microbiological profile and gingival inflammation susceptibility [8], and periodic controls were planned for her, first every six months and once a year later on.

## 7. Discussion

The transpalatal bar appliance (TPA) is a fixed appliance widely used to change the position of the molars in the three dimensions of space, to maintain the transversal width and provide anchorage when placed passive. Although there have been many attempts to improve and change its original design, the transpalatal bar more commonly used is the one designed by Goshgarian [2], consisting in a rigid wire or bar connecting the bands of both first maxillary molars. It is usually constructed with a loop in the middle that can be oriented both mesially or distally.

Another version of the TPA is the one with an acrylic button (which has sometimes been named as vertical holding appliance or VHA) [9] that is supposed to use the tongue pressure to restrain the normal descent of molars during the orthodontic treatment and to be beneficial in controlling the vertical development of the maxillary molars during their eruption. Wise et al. [10] found a reduction of 0.20 mm on the first maxillary molar's eruption, and DeBerardinis et al. [9] reported less posterior control and more open bite effect when using tip back ends plus high-pull headgear [11] compared with the group that was treated with VHA.

Other variations of the traditional transpalatal bar are the Quad-Helix [12], Burstone's lingual arches [13], and Zachrisson's type transpalatal bar (ZTPB) [14]. This last one was compared with the traditional Goshgarian transpalatal bar by Gündüz et al. in 2003 [2]. They found that ZTPB created lower contractive horizontal forces and that the lower load-deflection rate avoided the need for reactivation during derotation, and less amount of compensation was needed, compared to the greater moments of rotation that the classic Goshgarian type created.

TPA has been traditionally used as a soldered, passive appliance in order to stablish and maintain the transverse distance and provide anchorage [15], control the upper molar's eruption, prevent molars from rotation and buccolingual tipping [16], and have retention after rapid maxillary expansion and space maintenance in general. When the TPA is removable, it is a more versatile appliance, as the professional can activate it in order to obtain a wider variety of clinically useful movements in the 3 planes of space, such as move and rotate maxillary molars [17], uni- and bilateral derotation of the molars, changing the palatal arch form (achieving expansion or constriction) in order to correct unilateral crossbites, change the torque of the molars by activating the inserts, and provide buccal root torque of upper molars. However, we must be aware of the side effects [18] caused by the reactive forces of the movements we want to achieve, as the rigidity of the material and the fixed length of the transpalatal wire can have a constriction effect difficult to anticipate [16].

Although long used as a maximum anchorage device, TPA has been suggested to be a medium anchorage device; however, most of the studies concerning this data have tested it using finite element analysis or typodonts [19]. Bobak et al. [20] found that molars subjected to mesial forces showed periodontal stress (which is been used as a proof for movement in teeth) and that TPA only reduced it in a 1%. However, Kojima and Fukui [21] proved that, although TPA had no effect controlling the mesial tip or movement of the molars, it effectively prevented the rotation and transverse movement of the teeth that are created by mesial forces.

With the current widespread use of bone anchorage devices, which provide absolute anchorage, it has become even more important to know which of our classical devices are able to provide sufficient amount of anchorage to consider them or not as a treatment option. It seems not logical to choose a more expensive and complex device to perform the tasks that can be easily done with traditional and more affordable mechanics, until other methods would provide effective total inhibition of tooth movement [22].

## 8. Summary and Conclusions

Double transpalatal arches on both first and second maxillary molars are a successful appliance in order to control the posterior sectors and improve the torsion of the molars. They allow the professional to gain overbite instead of losing it as may happen with other techniques and avoid the Wilson curve to enlarge, obtaining a more stable occlusion without the need for extra help from bone anchorage.
